# Molecular dynamics study of ferroelectric domain nucleation and domain switching dynamics

**DOI:** 10.1038/s41598-017-01002-0

**Published:** 2017-04-11

**Authors:** Vishal Boddu, Florian Endres, Paul Steinmann

**Affiliations:** Chair of Applied Mechanics, University of Erlangen-Nuremberg, Egerlandstrasse 5, Erlangen, 91058 Germany

## Abstract

Ferroelectric materials contain domains of ordered electric dipoles, separated by domain walls, that can undergo polarisation switching under externally applied electric fields. The domain switching dynamics in ferroelectric materials plays an essential role in their application to electronic and electro-optic de- vices. Previous studies suggest that the switching occurs largely through domain wall motion which is explained from the viewpoint of statistical physics on surface growth as the behaviour of a pinned elas- tic interface. We perform molecular dynamics simulations to investigate the domain switching process and quantitatively estimate the switching speed of anti-parallel 180° domains in ferroelectric, tetragonal BaTiO_3_ perfect single crystals at room temperature using the core-shell model. We observe an unprece- dented, non-linear increase in the domain switching speed caused by the nucleation of new domains within the switching domain. We determine the strength of the electric field to evoke nucleation of new domains and show that the nucleated domains diffuse into nearby favourable domains when the electric field is removed. Furthermore, we discuss the prominence of domain nucleations during ferroelectric switching.

## Introduction

Ferroelectric materials are indispensable in a countless number of industrial and scientific applications due to their dielectric, piezoelectric, pyroelectric, electro-optic and electrical hysteresis properties^1^. The electrical hysteresis loops are characteristic of ferroelectric materials as they possess spontaneous electric polarisation, in a definite range of temperature, that can be switched to two or more stable states by application of an external electric field. This change in the polarisation direction is remnant if the electric field is sufficiently high. The critical strength of an electric field in order to change the direction of the polarisation permanently is the coercive field. The coercive field is material-dependent and is influenced by defects, doping, stoichiometry and illumination.

Closed regions where the dipole moments have the same directions are the ferroelectric domains. The interface between two domains is the domain wall. The domain structure and its evolution under external electric fields are direct consequences of polarisation switching processes. When an electric field is applied to a sample, domains with polarisation oriented along the field direction become energetically more favourable. Domains with such orientation are the growing domains. They grow at the expense of their neighbours, switching or reversal domains, through the propagation of existing domain walls and/or by the formation and growth of new domains.

180° domain wall motion and domain switching dynamics in single crystals have been studied experimentally^2–7^ and theoretically^8–10^. Experimentally, the domain wall motion is suggested as a non-linear dynamic process resulting from competition between elastic and pinning forces^6^. Nucleation and growth at the domain wall have been observed and analysed in detail^8^. It is suggested that the typical ferroelectric switching is largely governed by a simple, universal mechanism of intrinsic domain wall motion and that, even in the absence of defects, the electric field dependence of the domain wall speed can be described with a non-linear creep-like region and a depinning-like region^10^.

Ferroelectric domain walls, at temperatures (*T*) close to 0 K remain strongly pinned by local disorders and do not move until the applied electric field (*E*) crosses a critical value *E*_*C*0_. When *E* ≥ *E*_*C*0_, the domain wall experiences a pinning-depinning transition and starts to move with a finite speed (*v*). At finite temperatures, however, thermal activation enables a non-linear dynamic response for fields even below the critical value *E*_*C*0_. The domain wall motion at finite temperatures under relatively weak electric fields can be described by a creep process^5, 6, 10^:

1 $${\rm{\nu }}\propto \exp [-\frac{U}{{k}_{B}T}{(\frac{{E}_{C0}}{E})}^{\mu }],$$

where *U* is a characteristic energy barrier, *k*_*B*_ is Boltzmann’s constant, *E*_*C*0_ is a critical field at which depinning occurs at 0 K and *μ* is the dynamical exponent determined by the nature of the defects in the material sample. Equation 1 can be seen as a generalized formulation of the Merz’s law^2, 10^,

2 $${\rm{\nu }}\propto \exp [-\frac{{E}_{a}}{E}],$$

where *E*_*a*_ = *UE*_*C*0_/(*k*_*B*_ *T*) is the activation field and *μ* = 1.

Due to rich and pronounced finite size effects, ferroelectric behaviour needs to be probed and understood at different length scales. There have been efforts to develop ferroelectric material models at the macroscale, using finite element methods based on non-linear continuum mechanics, and at the mesoscale, using phase field methods based on the Landau-Devonshire theory of phase transition^11–13^. However, such models essentially rely on empirical parameters and thus do not allow re-examination of assumptions in existing theories of domain switching dynamics. Furthermore, phase field methods need certain physical parameters, such as domain wall energies, domain wall thickness and domain wall speed etc, which are usually computed by atomistic simulations^14^.

At present, full first-principles calculations of ferroelectric crystals are limited to a few hundred atoms per supercell at zero Kelvin. This has stimulated interest in the development and use of effective atomistic models, from which a portion of the degrees of freedom have been integrated out^15^. Atomistic models of perovskite ferroelectrics, such as isotropic and anisotropic core-shell models^16^, approaches based on effective Hamiltonians^17, 18^ and bond-valence models^19^, have been developed and shown to be suitable to probe a certain range of length and time scales that are currently beyond the reach of first-principles calculations. At the atomistic length scale the electric dipole of a single unit cell divided by its volume gives the electric polarisation of the unit cell. Thereby using molecular dynamics the domain switching processes can be studied with atomistic detail by computing the polarisation of each unit cell in the simulation system at any instant of time.

While domain nucleations inside the reversal domain (as opposed to nucleations at the domain wall) have been observed in epitaxial Pb(Zr,Ti)O_3_ and BaTiO_3_ capacitors through experiments^20, 21^, atomistic simulations with domain nucleations have not been observed or reported, and accounted for. We perform core-shell molecular dynamics simulations of domain switching processes in ferroelectric, tetragonal BaTiO_3_ single crystals (see Methods). In particular we investigate the switching processes and the domain wall movement in uncharged anti-parallel 180° domains. We apply a range of electric fields and identify the strength of the electric field (*E*_*N*_) necessary to evoke domain nucleation inside a domain. We find that the ferroelectric switching is characterized not just by the domain wall motion but more importantly by the nucleations and growth of new domains within the reversal domain. We explain the behaviour of domain switching speed in response to the applied electric field.

## Results and Discussion

**Figure 1:**
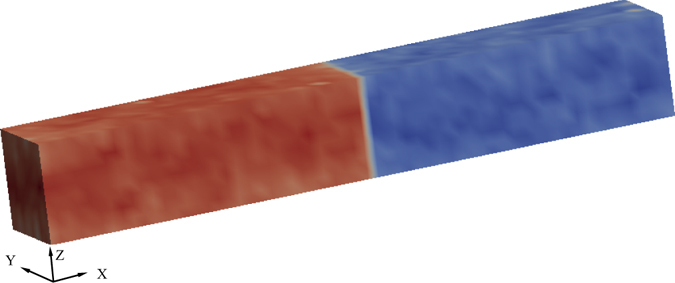
Schematic representation of the uncharged anti-parallel domains. 180° domain wall separating the growing domain and the reversal domain, coloured in red and blue respectively. The domains are anti-parallel in terms of their polarisation directions.

To investigate the ferroelectric switching processes in BaTiO_3_ single crystals, we consider a set-up of anti-parallel domains under periodic boundary condition separated by 180° domain walls as shown in Fig. 1. The growing and the reversal domains are shown in red and blue colours, respectively. The growing domain has its polarisation along the +*Z* direction and the reversal domain along the −*Z* direction. The growing domain, as its name suggests, grows at the expense of reversal domain in the presence of an electric field energetically favouring the growing domain. In an atomistic simulation such anti-parallel domains set-up can be created by applying anti-parallel electric fields. Such poling electric fields are removed when the anti-parallel domain set-up is deemed stable. In other words, both the growing and the reversal domains undergo an equilibration process after which the domain walls remain stationary in the absence of external electric fields. It is imperative that the domains created by poling electric fields are stable (see Methods).

**Figure 2:**
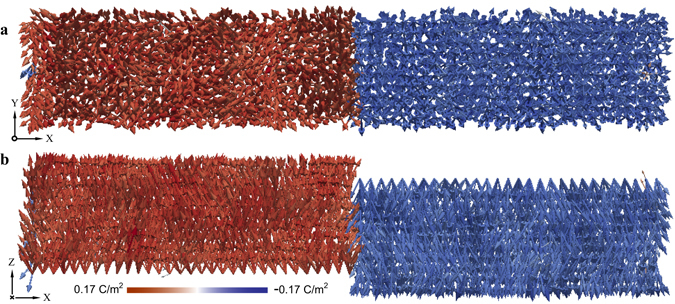
Polarization of unit cells after equilibration of uncharged anti-parallel domains. Each arrow represents polarisation of a single unit cell inside the fully equilibrated, periodic 60 × 10 × 10 supercell system. Arrows in red and blue indicate a positive *Z* component and a negative *Z* component, respectively. (**a** and **b)**, parallel projection of polarisation arrows onto the *XY* and the *XZ* plane, respectively.

After the anti-parallel domains set-up is achieved an external electric field is applied along the +*Z* direction, favouring the growing domain. The ferroelectric switching process is then studied under various external electric fields. To visualize the switching processes, a fixed colour map with red to blue gradation is employed to indicate the *Z* component of the polarisation of each unit cell in the system, as shown in Fig. 2.

### Weak electric fields

**Figure 3:**
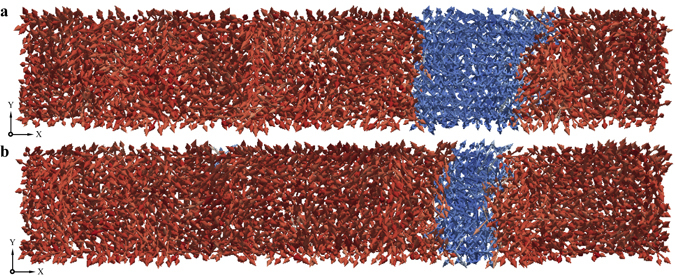
Domain pattern revealing propagation of domain walls. (**a**) Under an electric field of 20 MV/m, the domain patterns (**a** and **b**), at *t* = 55.4 ps and *t* = 77.2 ps respectively, reveal clearly that the switching process continues to be governed by the propagation of the domain walls. The domain walls approach closer to each other and merge producing a single domain after around *t* = 89 ps (not shown in the figure).

When the magnitude of the externally applied electric field is around or below 20 MV/m (in the case of BaTiO_3_ single crystals), the growing domain increases steadily and in a quantifiable manner. This is also to say that the domain walls gradually move into the reversal domain. The domain patterns obtained from the atomistic simulations at various instants during the ferroelectric switching process are shown in Fig. 3. Under such *weak* external electric fields, the ferroelectric switching occurs through a clear domain wall propagation. The domain walls approach closer to each other and would eventually merger producing a single domain with polarisation along +*Z*. Nucleation of new domains inside the reversal domain is not observed.

### Strong electric fields

**Figure 4:**
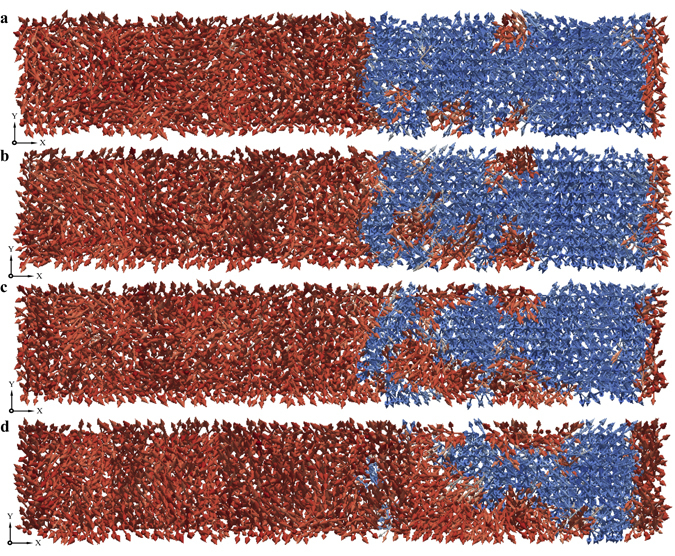
Domain pattern revealing nucleation of new domains. (**a**) At *t* = 4.8 ps nucleation of new domains inside reversal domain are observed under the field of 30 MV/m. (**b** and **c**) Domain patterns at *t* = 6.2 ps and *t* = 7.2 ps reveal that the nucleated domains grow in size (**d**), at *t* = 8.2 ps show that the some of the nucleated domains merge into nearby growing domain. After around *t* = 15.3 ps the system has completely switched which is not illustrated in the figure.

When the magnitude of the externally applied electric field is around or above 25 MV/m (in the case of BaTiO_3_ single crystals), the ferroelectric switching process is significantly different. The switching process under strong electric fields is dominated by nucleation of new domains in the reversal domain. Though the propagation of domain walls is identifiable, the contribution of domain wall propagation towards ferroelectric switching is considerably low. The domain patterns obtained from the atomistic simulations at various instants during the ferroelectric switching process under strong electric fields are shown in Fig. 4. Figure 4a shows four domain nucleations inside the reversal domain.

Two important inferences can be drawn upon studying the ferroelectric switching process under strong electric fields. First, the nucleation of new domains in the reversal domain appear primarily in the early stages of the switching process. Second, the nucleation of new domains create new domain walls which would further propagate through the reversal domain contributing to the switching process. Eventually however, apparent from Fig. 4d, the nucleated domains merge leaving behind fewer domain walls (or lesser domain wall area). The switching process occurs through propagation of the domain walls thereafter.

**Figure 5:**
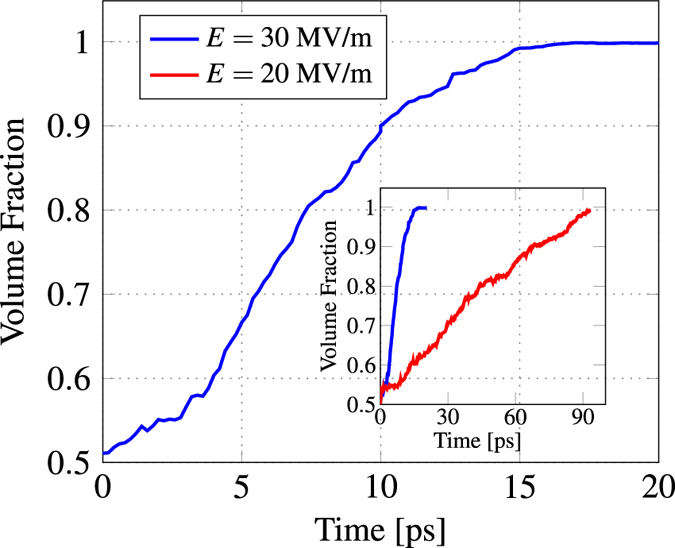
Evolution of the growing domain with time. The time-dependent volume fraction of the growing domain is computed for every 0.1 ps during the switching process under a strong electric field, *E* = 30 MV/m. After equilibration when the time is set to zero, due to thermal fluctuations, it is possible that the volume fraction of growing domain is very close but not exactly equal to 0.5. The inset shows the comparison with the time-dependent volume fractions under a weak electric field, *E* = 20 MV/m.

Since the switching processes under weak and strong electric fields are significantly different, one expects that the switching kinetics are also different. The switching kinetics can be obtained by pursuing the time-dependent evolution of the fraction of growing domains (or reversal domains) or by pursuing the change in overall polarisation during the switching processes. Figure 5 shows the evolution of the fraction of growing domains under a strong electric field, *E* = 30 MV/m. The initial stages essentially include overcoming the activation energy required for the nucleation of new domains. Nucleated domains and their growth are then responsible for speeding up the switching process until they merge. After most of the nucleated domains merge, the switching process slows down as the subsequent switching occurs primarily through propagation of the domain walls. On the contrary, the switching kinetics under a weak electric field remain almost linear.

It can be observed that the switching kinetics under a strong field resembles that of mono-domain switching. Experimentally, the switching kinetics of mono-domains have been explained using the Kolmogorov-Avrami-Ishibashi (KAI) model^22^. The KAI model is based on the classical approach of nucleation and subsequent growth of reversed domains^23^. It describes the time-dependent normalized change in polarisation as a Lorentzian function similar to the time-dependent evolution of the fraction of growing domains obtained in Fig. 5. The similarity in the kinetic behaviour is due to the similarity in switching processes indicating the prominence of domain nucleations during ferroelectric switching.

The emergence and the number of domain nucleations increases with an increase in the electric field strength. From the experimental point of view, the emergence of domain nucleations could mean that a large jump in the switching current is observed. Overall, the nucleation of new domains along with the creation of new domain walls drastically reduces the total switching time under strong electric fields.

### Diffusion of nucleated domains

In an earlier theoretical study through atomistic simulations, it is concluded that the ferroelectric domain walls do not exhibit significant intrinsic inertial response^9^. In other words, the evolution of domain pattern revealed that the domain walls stop moving when the electric field is turned off and show that the domain wall speed is solely determined by the strength of the electric field at any given time. The simulations performed in this study are in agreement with the above under weak electric fields; on removal of the electric field the domain walls remained stationary. However, the evolution of domain pattern upon application and removal of strong electric fields is slightly different. This is due to the sustained nucleations of new domains.

**Figure 6:**
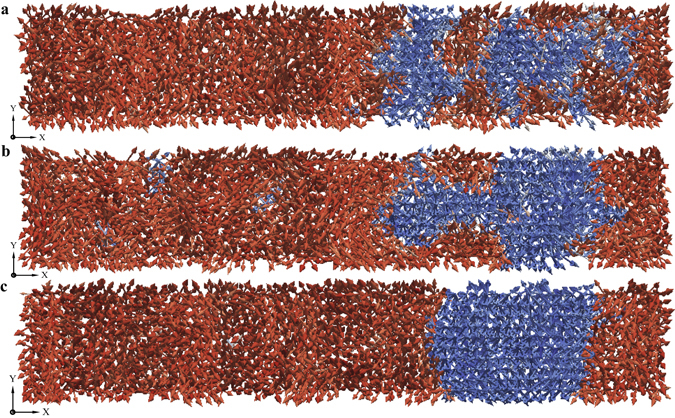
Domain pattern revealing domain diffusion. (**a**) At *t* = 9.2 ps the domain pattern shows nucleated domains under a field of 35 MV/m, exactly at this instance the electric field is removed. (**b**) At *t* = 21.2 ps the nucleated domains appear to diffuse in the absence of the electric field. (**c**) By *t* = 51.2 ps the nucleated domains have completely diffused into the growing domain and the resulting domain pattern continues to remain unchanged.

The nucleated domains grow as long as the (strong) electric field is not removed. When the electric field is removed, the nucleated domains diffuse into the nearby nucleated domain or into the growing domain so as to reduce the total surface area of the domain walls as shown in Fig. 6. This shows that while there is no inertial response, there is an intrinsic response from the nucleated domains. The intrinsic response is such that it minimizes the domain wall energy in the system. Flat domain walls, as seen in Fig. 6c, indicate that the total interface area between the domains is minimized.

### Domain switching speed

**Figure 7:**
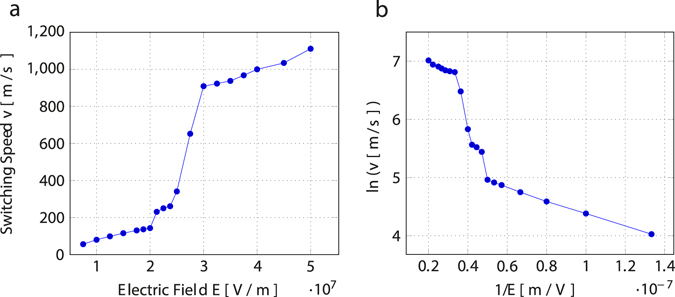
Average domain switching speed at various electric field values. (**a**) Plot of switching speed (v) versus electric field (E) showing a jump around 25 MV/m. (**b**) Plot of ln(v) versus 1/E shows a negative linear relation describing a creep process at low electric field values.

To account for the influence of domain nucleations on the switching process under strong electric fields, a better descriptor of the switching process as opposed to the domain wall speed is necessary. Figure 7 shows the switching speed, which is defined as the ratio of transversal length of the reversal domain to the total switching time, under a range of electric fields. A large jump in the switching speed is seen between 20–30 MV/m. We attribute this jump in switching speed to the nucleation of new domains and their growth.

It is known that the density of domain nucleations increases with the applied electric field. We have not observed domain nucleations in simulations performed with the reversal domain of less than 20 unit cells (transversal length). Moreover, the critical electric field of 25 MV/m required to observe nucleations is specific to the reversal domain (30 unit cells) employed in this study. We hypothesize that in atomistic simulations of ferroelectric switching under weak electric fields, nucleation of new domains are unlikely to be observed due to the limited size of the reversal domain. Perhaps, it is possible to observe domain nucleations under a weaker critical field if one considers larger reversal domains for atomistic simulations. In other words the nucleation of new domains has pronounced effects on the polarisation switching dynamics in larger reversal domains. Further atomistic simulations are needed to study the size effects of the reversal domain on the critical field for nucleation of new domains.

Previously reported experimental values of the electric field to cause dielectric breakdown in BaTiO_3_ single crystals are in the range of 50–365 MV/m^24–27^. Nucleation and growth of ferroelectric domains have been studied up to field strengths of at least 45 MV/m in bulk BaTiO_3_ single crystals and 1.3 GV/m in thin films^3, 25^. In this study, we observed domain nucleations at a much weaker field strength of 25 MV/m. We therefore rule out the possibility that the domain nucleations observed with our atomistic simulations are a consequence of a dielectric breakdown.

## Conclusions

The domain switching speed in ferroelectric BaTiO_3_ single crystal under various electric fields are computed using molecular dynamics simulations. The domain wall motion under weak electric fields follows Merz’s law which can be generalized as a creep-like process. However, a large jump in the switching speed has been observed at around 25 MV/m. This is attributed to the emergence of domain nucleations and domain walls generated thereby. We establish that the domain nucleations can be observed, even within a limited size of the reversal domain, under strong electric fields using atomistic simulations. If the electric field is removed, the nucleated domains diffuse so as to minimize the total domain wall area. We reason that in atomistic simulations of ferroelectric switching under electric fields less than the established *E*_*N*_ nucleation of new domains are unlikely to be observed due to the limited size of the reversal domain.

## Methods

### Molecular dynamics simulations

We carried out isothermal-isobaric (NPT) molecular dynamics simulations to study the ferroelectric switching process in tetragonal BaTiO_3_ single crystals at atmospheric pressure and close to room temperature (310 K). We use the isotropic, an-harmonic core-shell model potential for BaTiO_3_ (presented below) that has been fitted to results of first-principles density functional theory calculations with a modified generalized gradient approximation^28^. All the simulations are performed with 60 × 10 × 10 unit cells under periodic boundary conditions. The thermostat and barostat relaxation times are both set to 0.1 ps. All shells are assigned a mass of 2 a.u. and their respective cores are assigned the remaining atomic mass^29^. The times step used is 0.4 fs. The growing and the reversal domains are generated through a two step process. The unit cells in the system are first poled using an external electric field for 4 ps. The left half of the system is poled in the +*Z* direction and the right half is poled in the −*Z* direction. The external field is then removed and the system is equilibrated for another 4 ps. Now, if the system is kept under no external bias, the growing domain and the reversal domain maintain the same average polarisation and the domain walls remain stationary. This ensures the stability of both the domains and the domain walls generated thereby. After the domains are prepared, the time (*t*) is set to zero and an external electric field is applied in the +*Z* direction. We then follow the switching process by computing the polarisation of each lattice unit cell in the system. In this study, we compute the polarisation of a unit cell using the core-shell charges^30, 31^.

### The core-shell model

The core-shell model has been used in molecular statics and molecular dynamics simulations to gain insights into the properties of ferroelectric materials^32–35^. In the core-shell model every ion is represented in terms of a charged (atom) core and a charged (electron) shell, linked by a harmonic or an-harmonic spring. This introduces electronic Polarizability in the ions. In the conventional core-shell model the shells are assumed to be massless. Because the shells have no mass, they respond instantaneously to the motion of the cores i.e., at any instant during a molecular dynamics simulation the shells are repositioned so as to be in the minimum potential energy configuration with respect to the current cores’ configuration. In this work we adopt an alternative approach by assigning a small mass to the shells and treating their motion dynamically similar to that of the cores since this approach has been shown to be computationally more efficient^36^. We therefore treat cores and shells as point mass particles interacting classically through potentials.

### BaTiO_3_ isotropic, an-harmonic core-shell model potential

The interaction between cores and shells is described by three different interaction potentials. The interaction between the core and the shell of an atom is treated exclusively by an isotropic, an-harmonic spring with the potential parameters *k*_2_ and *k*_4_,

3 $${\varphi }^{S}(|{{\bf{r}}}_{ij}|)=\frac{1}{2}{k}_{2}{|{{\bf{r}}}_{ij}|}^{2}+\frac{1}{24}{k}_{4}{|{{\bf{r}}}_{ij}|}^{4}.$$

The position vectors of particles *i* and *j* are given by **r**_*i*_ and **r**_*j*_, respectively. The norm of the distance vector between two interacting particles *i* and *j* is given by |**r**_*ij*_| = |**r**_*j*_ − **r**_*i*_|.

The interactions between shells of different atoms, which describe the electron cloud repulsion and the Van der Waals attraction, are modelled using the Buckingham potential,

4 $${\varphi }^{B}(|{{\bf{r}}}_{ij}|)={A}\,\exp (-\frac{|{{\bf{r}}}_{ij}|}{\rho })-\frac{c}{{|{{\bf{r}}}_{ij}|}^{6}}.$$

The variables *A*, *ρ*, and *c* denote potential parameters.

Finally, the electrostatic interactions among the charged particles viz., cores and shells are considered through the Coulomb potential,

5 $${\varphi }_{i}^{C}=\frac{1}{4\pi {\varepsilon }_{0}}\sum _{j\ne i}^{N}\frac{{q}_{i}{q}_{j}}{|{{\bf{r}}}_{ij}|}.$$

**Figure 8:**
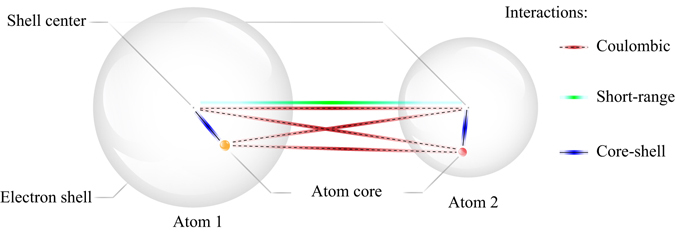
Schematic representation of the interactions in the core-shell model. Each atom is treated as having two components: a charged shell and a charged core connected by a nonlinear spring.

The particle *i*’s charge is given by *q*_*i*_, *ε*_0_ is the electric permittivity of free space and the total number of particles is denoted by *N*. Note that there is no electrostatic interactions between an atom’s core and its respective shell in the core-shell model. Figure 8 schematically illustrates the interaction between two atoms using the core-shell model.

**Figure 9:**
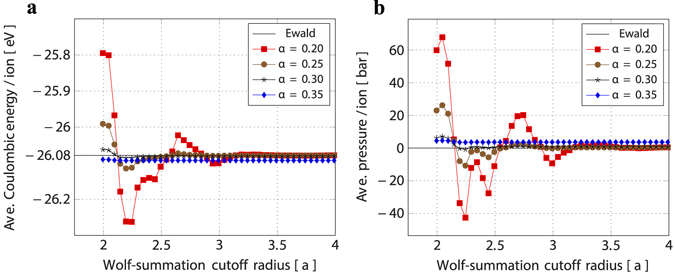
Effect of damping coefficient and cutoff radius in computing Coulombic energy and pressure. (**a**) Ewald summation is used as a standard for comparison of accumulated Coulomb energy per ion. (**b**) The pressure inside the system is sensitive to the damping coefficient. Computations are performed using a system of periodic 10 × 10 × 10 unit cells of rhombohedral BaTiO_3_ at 0 K.

Accumulation of the electrostatic interactions is one of the most computationally demanding tasks because of the long-range character of the Coulomb potential. Conventionally, Ewald summation is used to accumulate electrostatic interactions in a system with periodic boundary conditions with high accuracy and precision. For large system sizes, however, the Ewald summation method suffers from high computational costs. We therefore substitute the Coulomb potential by the Wolf summation approach,

6 $$\begin{array}{rcl}{\varphi }_{i}^{Wolf} & = & \frac{1}{4\pi {\varepsilon }_{0}}\sum _{\begin{array}{c}j\ne i\\ |{{\bf{r}}}_{ij}| < Rc\end{array}}[\frac{{q}_{i}{q}_{j}{\rm{erfc}}(\alpha |{{\bf{r}}}_{ij}|)}{|{{\bf{r}}}_{ij}|}-\mathop{\mathrm{lim}}\limits_{|{{\bf{r}}}_{ij}|\to {R}_{c}}\{\frac{{q}_{i}{q}_{j}{\rm{erfc}}(\alpha |{{\bf{r}}}_{ij}|)}{|{{\bf{r}}}_{ij}|}\}]\\  &  & -\frac{1}{2}[\frac{{\rm{erfc}}(\alpha {R}_{c})}{2{R}_{c}}+\frac{\alpha }{{\pi }^{\frac{1}{2}}}]\sum _{i=1}^{N}{q}_{i}^{2},\end{array}$$

which involves a simple modification to the direct pairwise sum but scales approximately linearly with the system size^37, 38^. In equation 6, the complementary error function reads as erfc $(x)=1-(\mathrm{2/}\sqrt{\pi }){\int }_{0}^{x}{e}^{-{\tau }^{2}}$, *α* is the damping coefficient and *R*_*c*_ is the cutoff radius to truncate the pairwise sum. The damping coefficient *α* and cutoff radius *R*_*c*_ must be carefully chosen and calibrated such that the accumulated electrostatic interactions equals with that of the Ewald summation and that there is no artificial pressure developed in the system. To this end, we have studied the effect of the damping coefficient and the cutoff radius on the average Coulombic energy and pressure per ion, captured in the Fig. 9. A higher damping coefficient allows the use of a lower cutoff radius with the consequence of artificial pressure built-up in the simulation system.

It is worth mentioning that the only other molecular dynamics study to quantitatively estimate domain wall speed, to the best of our knowledge, was conducted using the bond-valence model potential^10, 19^ as opposed to the core-shell model potential, in PbTiO_3_ perfect single crystals. The potential parameters used in this study are taken from ref. 23. They are obtained from performing least square minimization of the energy differences in the potential energy surface between the core-shell model and first-principles density functional theory calculations. The potential parameters are shown in Table 1.

**Table 1:** BaTiO_3_ core-shell model parameters used in this study^28^.

Atom	Core charge	Shell charge	*k*_2_	*k*_4_
	(|*e*|)	(|*e*|)	(eV/Å^2^)	(eV/Å^4^)
Ba	5.042	−2.870	298.51	0.0
Ti	4.616	−1.544	306.14	500.0
O	0.970	−2.718	36.93	5000.0
Short-range	A (eV)	*ρ* (Å)	c (eV Å^6^)	
Ba-O	7149.81	0.3019	0.0	
Ti-O	7220.27	0.2303	0.0	
O-O	3719.60	0.3408	597.17	

### Reproducing BaTiO_3_ phase transition sequence and temperatures using the core-shell model

**Figure 10:**
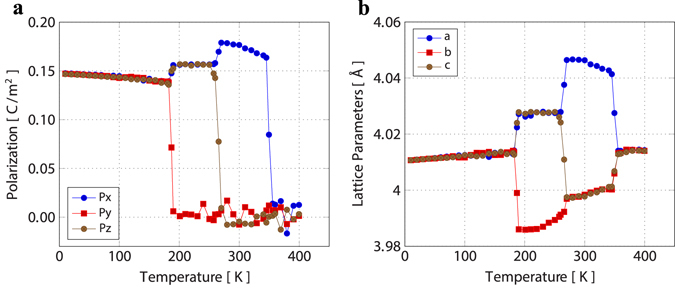
Phase transition sequence and temperatures for BaTiO_3_. (**a**) Average lattice constants and (**b**), average polarisation along the the three lattice directions as function of temperature. Core-shell molecular dynamics is performed with a system of periodic 10 × 10 × 10 unit cells^28^.

The core-shell model reproduces the correct sequence of phase transitions: Rhombohedral (0–190 K) → Orthorhombic (190–270 K) → Tetragonal (270–350 K) → Cubic, shown in Fig. 10. The phase transition temperatures are also in close agreement with a slight underestimation of the Tetragonal to Cubic transition when compared with experimental values^28^.
